# Using the Zebrafish as a Genetic Model to Study Erythropoiesis

**DOI:** 10.3390/ijms221910475

**Published:** 2021-09-28

**Authors:** Yuhan Zhang, Mengying Chen, Caiyong Chen

**Affiliations:** MOE Key Laboratory of Biosystems Homeostasis & Protection, College of Life Sciences, Zhejiang University, Hangzhou 310058, China; 3150105228@zju.edu.cn (Y.Z.); 11507044@zju.edu.cn (M.C.)

**Keywords:** erythropoiesis, zebrafish, iron metabolism, heme homeostasis, anemias

## Abstract

Vertebrates generate mature red blood cells (RBCs) via a highly regulated, multistep process called erythropoiesis. Erythropoiesis involves synthesis of heme and hemoglobin, clearance of the nuclei and other organelles, and remodeling of the plasma membrane, and these processes are exquisitely coordinated by specific regulatory factors including transcriptional factors and signaling molecules. Defects in erythropoiesis can lead to blood disorders such as congenital dyserythropoietic anemias, Diamond–Blackfan anemias, sideroblastic anemias, myelodysplastic syndrome, and porphyria. The molecular mechanisms of erythropoiesis are highly conserved between fish and mammals, and the zebrafish (*Danio rerio*) has provided a powerful genetic model for studying erythropoiesis. Studies in zebrafish have yielded important insights into RBC development and established a number of models for human blood diseases. Here, we focus on latest discoveries of the molecular processes and mechanisms regulating zebrafish erythropoiesis and summarize newly established zebrafish models of human anemias.

## 1. Introduction

Erythropoiesis is a complex process in which the erythroid progenitors undergo multiple differentiation events to become mature red blood cells (RBCs). The common ancestors of erythroid cells, the megakaryocyte–erythroid progenitors (MEPs), are derived from hematopoietic stem cells (HSCs). Early stages of erythropoiesis involve the proliferation and differentiation of erythroid progenitors including the burst forming unit-erythroid (BFU-E) and the colony forming unit-erythroid (CFU-E), in an erythropoietin (EPO)-dependent manner. CFU-Es mature through several morphologically distinguishable stages, including proerythroblasts and basophilic, polychromatic, and orthochromatic erythroblasts, and develop into biconcave-shaped reticulocytes [1,2]. The differentiation process between CFU-Es and reticulocytes, called terminal erythropoiesis, involves production of vast amounts of hemoglobin, nuclear condensation and extrusion, membrane remodeling, cell cycle exit, and clearance of all cellular organelles [3,4,5]. During terminal differentiation, the intermediate erythroid precursors become less responsive to EPO, and iron instead plays an important regulatory role [6,7]. Each step of erythropoiesis is exquisitely regulated by specific factors especially transcription factors and signaling molecules. For example, the transcription factors TAL1, LMO2, and GATA2 are necessary for the establishment of erythroid lineage, while JAK-STAT and BMP-SMAD signaling pathways regulate the expansion and differentiation of erythroid progenitors [8,9]. Defects in erythropoiesis may lead to many types of anemias, such as β-thalassemia, microcytic hypochromic anemia, congenital dyserythropoietic anemias (CDAs), Diamond–Blackfan anemias (DBAs), and myelodysplastic syndrome (MDS). 

Studies on genetic models have provided many important insights into the fundamental understanding of erythropoiesis. The model organisms *Saccharomyces cerevisiae* and *Caenorhabditis elegans* are widely used to investigate iron and heme trafficking pathways. The mouse as a mammal has well-defined hematopoietic cell lineages in the fetal liver and bone marrow, and it provides an excellent model for tracing the lineages of progenitor cells and for analyzing the cell–cell interaction within the complex hematopoietic niche. For example, the multipotent nature of HSCs was first revealed by studies in mice. 

The zebrafish has emerged as an important genetic model for studying erythropoiesis. It has several advantages over other model organisms, including rapid development, external fertilization, optically transparent embryos, large number of progeny, similar erythropoiesis process to mammals, and well-established genetic techniques including morpholino-mediated knockdown, CRISPR-mediated knockout, knock-in, and mutagenesis screening [10,11]. Gene silencing in zebrafish embryos with morpholinos enables rapid gene function analysis in vivo, and the large progeny size facilitates forward genetic screens and chemical screens. In addition, zebrafish embryos can tolerate extreme anemia, making it feasible to analyze the physiological function of essential erythroid genes in vivo. Thus, the zebrafish has been used not only to study the cellular and molecular mechanisms of red cell development but also as a powerful tool for large-scale compound screens in order to identify new therapeutic drugs for blood diseases [12,13,14].

## 2. Zebrafish Erythropoiesis

**Figure 1:**
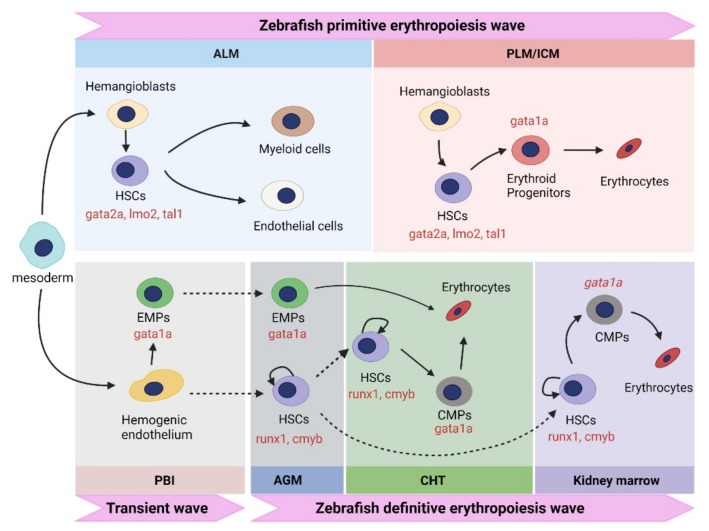
Primitive and definitive erythropoiesis in zebrafish. Primitive erythropoiesis is shown on the top. The transient wave and definitive erythropoiesis are shown at the bottom. Transcription factors critical to erythropoiesis are shown in red. Dotted arrows represent migration, while solid arrows stand for differentiation or self-renewal. HSC, hematopoietic stem cell; ALM, anterior lateral mesoderm; PLM; the posterior lateral mesoderm; ICM, intermediate cell mass; EMPs, erythro-myeloid progenitors; CMP, common myeloid progenitors; PBI, posterior blood island; AGM, aorta–gonad–mesonephros; CHT, caudal hematopoietic tissues.

Zebrafish erythropoiesis comprises two successive waves, the primitive wave and the definitive wave. The primitive wave generates erythrocytes and macrophages for the development of early embryos, while the definitive wave generates definitive HSCs to maintain blood cell production throughout the zebrafish’s lifetime [15,16]. During primitive and definitive erythropoiesis, HSCs are derived from hemangioblast and hemogenic endothelium, respectively. HSCs are at the top of the hematopoietic hierarchy and may differentiate to several blood lineages including RBCs, megakaryocytes, myeloid cells (monocyte/macrophage and neutrophil), and lymphocytes [2]. In zebrafish, HSCs reside in a highly specialized anatomic location, known as the hematopoietic niche, which is composed of endothelial cells, stromal cells, primitive myeloid cells, and melanocytes. The cell-to-cell signaling in the hematopoietic niche is crucial for regulating the self-renewal and differentiation of HSCs. For more details on zebrafish HSCs and the hematopoietic niche, please refer to the previous reviews [1,17,18]. In this part, we mainly focus on the production of erythrocytes, including both primitive erythropoiesis and definitive erythropoiesis (Figure 1). 

### 2.1. Primitive Erythropoiesis in Zebrafish

In zebrafish, hematopoietic cells, as well as vascular endothelial cells, pronephros, and kidney, are generated from the ventral mesoderm. When zebrafish embryos develop to ~11 hours post fertilization (hpf), the primitive erythropoiesis starts at the anterior lateral mesoderm (ALM) and the posterior lateral mesoderm (PLM) where hemangioblasts give birth to HSCs. The hemangioblasts act as bi-potential progenitors of both erythroid cells and endothelial cells [19,20,21]. The intermediate cell mass (ICM), the functional equivalent of the extraembryonic blood islands of mammals, is derived from later-stage PLM. In PLM/ICM, HSCs differentiate into erythroid progenitors, which subsequently generates erythrocytes.

A number of transcription factors, including tal1, gata2a, and lmo2, are expressed in the ALM and PLM for the specification of HSCs into erythroid progenitors during primitive erythropoiesis. Tal1, also known as stem cell leukemia (scl), is a transcription factor with a helix–loop–helix structure required for both primitive and definitive erythropoiesis [22]. GATA2 has a key role in the maintenance of HSCs in mammals [23]. Zebrafish has two orthologs of mammalian GATA2, *gata2a* and *gata2b*, and they play distinct roles in erythropoiesis [24,25]. Lmo2 may interact with scl to control the transcription of erythropoiesis-related genes [26]. Another transcription factor crucial to primitive erythropoiesis is gata1, which contains two zinc finger domains [27]. GATA1 activates the expression of many erythroid genes including those encoding hemoglobin subunits, heme synthesis enzymes, and iron uptake proteins. Additionally, GATA1 may transcriptionally regulate genes involved in autophagy and exosomes to coordinate these processes with erythropoiesis [28,29,30].

### 2.2. Definitive Erythropoiesis in Zebrafish

The aorta–gonad–mesonephros (AGM), caudal hematopoietic tissue (CHT), and kidney marrow are definitive hematopoiesis sites in zebrafish. The definitive erythropoiesis in zebrafish may be subdivided into two waves. A transient wave takes place at the posterior blood island (PBI) where hemogenic endothelium differentiates into erythro-myeloid progenitors (EMPs). EMPs then migrate into the dorsal aorta at ~26 hpf and differentiate into erythrocytes in CHT at ~48 hpf. 

The second definitive wave starts when the hemogenic endothelium gives birth to HSCs in dorsal aorta. At ~26 hpf, the definitive HSCs first appear in the AGM, which is located between the dorsal aorta and the axial vein [31]. HSCs then migrate from the AGM to the pronephros via two discrete routes. The first route is through CHT and circulation (Figure 1). The endothelial-to-hematopoietic transition (EHT) takes place at the ventral wall of the dorsal aorta (VDA) where endothelial cells change their shape, egress into the subaortic space, and enter circulation via the axial vein [32]. HSCs express cluster of differentiation 41 (*cd41*), enter the blood circulation, and colonize the CHT at ~48 hpf [33]. From ~48 to 96 hpf, the nascent HSCs migrate to the CHT, followed by expansion and differentiation [33,34]. The expanded HSCs then migrate to the pronephros at ~4 days post fertilization (dpf), where they differentiate into common myeloid progenitors (CMPs) [33]. CMPs differentiate into MEPs and subsequently into erythroblasts in a gata1-dependent manner. Definitive erythrocytes populate the circulation at ~5 dpf [35]. The second migration route does not involve circulation. The HSCs derived from the AGM enter the pronephros via pronephric tubules [33].

In zebrafish, definitive HSCs express a number of marker genes, including runt-related transcription factor 1 (*runx1*), *cmyb*, and cluster of differentiation 41 (*cd41*) [34,36,37]. Runx1 is a transcription factor essential for the generation of HSCs from endothelial cells, as its deficiency impairs the initiation of EHT in zebrafish embryos [32]. HSCs expressing *runx1* first appear at ~24 hpf in the VDA, validating it as the initiation site of definitive erythropoiesis [38]. At ~26 hpf, *runx1^+^* HSCs originating from the dorsa aorta start to express *cmyb*, which is essential for the migration of HSCs [39]. Similar to that in the mouse, zebrafish cd41 serves as an early surface marker of HSCs in the dorsal aorta [33].

### 2.3. Comparison of Zebrafish Erythropoiesis with Mammalian Erythropoiesis 

The molecular and cellular pathways, as well as the regulatory mechanisms, of erythropoiesis are highly conserved between teleosts and mammals. Erythropoiesis in both zebrafish and humans involves two successive waves, the primitive wave and definitive wave. Additionally, the regulatory network of erythropoiesis is highly conserved between zebrafish and humans [40]. For instance, homologs of almost all transcription factors with important roles in mammalian erythropoiesis are present in zebrafish [41]. Due to the high conservation of erythropoiesis in vertebrates, mutations in erythropoietic genes commonly lead to similar phenotypes in zebrafish and humans [42].

Although zebrafish erythropoiesis closely resembles that of mammals, it is distinct from mammalian erythropoiesis in a few aspects. First, the anatomical sites for generating erythrocytes are different. In zebrafish, primitive erythropoiesis takes place in the ICM, whereas the extra-embryonic yolk sac is the location of primitive erythropoiesis in mammals. Similarly, definitive erythropoiesis takes place in the PBI, dorsal aorta, CHT, and the kidney marrow in zebrafish, whereas the mammalian definitive erythropoietic sites are the AGM, placenta, fetal liver, and the bone marrow. Second, unlike mammalian RBCs, mature erythrocytes in zebrafish have nuclei. While zebrafish erythroblasts also undergo substantial nuclear condensation, they do not extrude the nuclei.

## 3. Using Zebrafish to Study the Regulation of Erythropoiesis

Vertebrate erythropoiesis is controlled by complex regulatory networks and feedback mechanisms in a spatially and temporally coordinated manner. First, each step of erythroid differentiation is controlled by specific transcription factors such as TAL1, GATA2, LMO2, RUNX1, CMYB, and GATA1. Second, two classical signal transduction pathways, the EPO–JAK–STAT pathway and the BMP–SMAD pathway, are known to play essential roles in regulating erythropoiesis in vertebrates. These signaling pathways are strictly controlled during erythropoiesis. Erythroferrone (ERFE), a hormone discovered recently, coordinates erythropoiesis with iron metabolism via regulating the BMP–SMAD pathway [43,44]. Third, erythropoiesis is regulated by microRNAs and long noncoding RNAs (lncRNAs). A number of microRNAs (miRNAs), including miR-126, miR-144, miR-451, miR-26a, and miR-23a, have been reported to regulate gene expression during red cell development [45,46,47,48,49,50]. In addition, erythropoiesis is regulated by recruiter lncRNAs, including LncHSC-2, lincRNA-EPS, and alncRNA-EC7/Bloodlinc, and decoy lncRNAs, including lnc-DC and lnc-MC [51]. For more detailed pathways and mechanisms regulating vertebrate erythropoiesis, please refer to the previous reviews [1,41,52,53]. In this section, we summarize recent progress on the regulation of erythropoiesis with emphasis on the studies in zebrafish. 

### 3.1. Erythroid Transcription Factors and Their Regulation

GATA1 is a transcription factor critical for erythropoiesis. Both loss and overexpression of GATA1 impair red cell development, demonstrating the importance of GATA1 homeostasis during erythropoiesis [54,55]. Recent studies in zebrafish have gained new insights into the regulation of gata1 and demonstrated that gata1 can be regulated at transcriptional and post-transcriptional levels. The ten–eleven translocation (TET) is a protein family responsible for converting methylcytosine (5mC) into hydroxymethylcytosine (5hmC) which may lead to DNA demethylation [56,57]. Zebrafish has three TET family genes, *tet1*, *tet2*, and *tet3*, and all of them can catalyze the formation of 5hmC in zebrafish embryos [58]. *tet2* can activate the expression of lineage-specific genes such as *gata1*, *scl*, and *cmyb*, and thus plays a key role in erythropoiesis [58]. Knockdown of *tet2* in zebrafish embryos resulted in decreased 5hmC levels in the intermediate CpG promoters of *gata1*, *scl*, and *cmyb*, leading to reduced expression of these transcription factors and impaired erythropoiesis [58]. Hypoxia-inducible factors Hif1α and Hif3α play important roles in upregulating gata1 expression to promote erythropoiesis [59,60]. Hif1α and Hif3α bind to the hypoxia response elements (HREs) in the 3’ flanking region and promoter region of *gata1*, respectively [59,60]. Loss of these *hif* genes in zebrafish impaired erythropoiesis, as the consequence of inadequate activation of gata1 expression [59,60]. In addition to transcription activation, inhibitory mechanisms exist to prevent excessive erythropoiesis. For example, deficiency of *p2y12*, an ADP receptor crucial for purinergic signaling [61], resulted in excessive primitive erythropoiesis in zebrafish embryos characterized by increased expression of α-globin, β-globin, and *gata1* [62]. This phenotype was mainly attributed to enhanced expression of *gata1* as it was rescued by injection of a *gata1*-specific morpholino [62].

The expression of *gata1* is also controlled at post-transcriptional levels during erythropoiesis. Elavl1 is an RNA-binding protein that binds AU-rich elements in mRNAs. In zebrafish embryos, elavl1a was found to bind the 3’ UTR of *gata1* mRNA [63]. Silencing of *elavl1a* caused a dramatic reduction in *gata1* expression, leading to impaired erythropoiesis [63]. Further studies reveal that the activity of ELAVL1 is controlled by protein kinase C (PKC), which phosphorylates ELAVL1 on Ser219 and Ser316 and promotes the translocation of ELAVL1 from the nucleus to cytoplasm to stabilize mRNA targets [64]. Similar to the knockdown of *elavl1a*, PKC inhibitors impaired erythropoiesis in zebrafish embryos [64]. In addition, the gata1 protein may be degraded through a caspase-dependent mechanism. In zebrafish, infection-induced inflammasome can activate caspase 1, which leads to cleavage and degradation of gata1 and dyserythropoiesis [65]. Pharmacological inhibition of caspase 1 can rescue the anemic phenotype in *gata1*-deficient zebrafish, providing a potential therapeutic strategy to treat this erythropoietic disorder [56].

Recent studies also revealed the critical roles of several other transcription factors in zebrafish erythropoiesis. Functional analyses of Krüppel-like transcription factor (klf) genes in zebrafish embryos revealed important roles of klf3 and klf6a in regulating erythroid differentiation [66]. *Klf3* promoted erythropoiesis through inhibiting the expression of ferric-chelate reductase 1b (*frrs1b*), while *klf6a* acted in a distinct manner by negatively regulating the expression of *cdkn1a* to control the cell cycle [66]. Forkhead box O3 (FOXO3), a member of the forkhead family, is also found to play an important role in vertebrate erythropoiesis. Morpholino knockdown of *foxo3b* resulted in decreased expression of *gata1* as well as globin genes and defective erythropoiesis in zebrafish embryos [67]. Another transcription factor, etv7, regulates the expression of the zebrafish *lss* gene, which encodes lanosterol synthase, an enzyme in the cholesterol synthesis pathway [68]. Knockdown of zebrafish *etv7* led to reduced hemoglobin production in erythroblasts, and this phenotype could be rescued by injection of exogenous cholesterol [68]. Importantly, zebrafish has a unique advantage in the study of the function of etv7 in erythropoiesis because mice, unlike humans, lack this gene [68]. 

### 3.2. Signaling Pathways

The role of the EPO–JAK–STAT pathway in erythropoiesis is highly conserved in vertebrates. In zebrafish, both primitive and definitive erythropoiesis require epo [69]. Three splice variants of the *epo* gene, *epo-L1*, *epo-L2*, and *epo-S*, have been detected in zebrafish [70]. *epo-S* is mainly expressed in the kidney marrow, whereas *epo-L1* and *epo-L2* are expressed in the liver and heart [70]. It has been well established that epo signaling is regulated by hypoxia-induced factors [71,72]. KIT ligand (KITLG) is another cytokine that regulates proliferation and differentiation of erythroid cells by binding to its receptors [73]. Recent gain-of-function studies in zebrafish showed that expression of *kitlga* and *kitlgb* both enhanced erythroid cell expansion by cooperating with epo [74]. The function of Kitlga in erythropoiesis was also verified in ex vivo suspension cultures of zebrafish hematopoietic progenitor cells [74]. Additionally, lysophosphatidic acid (LPA) was shown to cooperate with EPO signaling to regulate erythropoiesis, and its receptors, LPA2 and LPA3, play opposing roles in this process [75]. In human K562 cells, knockdown of LPA2 enhanced erythropoiesis, whereas knockdown of LPA3 inhibited RBC differentiation [75]. Consistently, hemoglobin expression in zebrafish embryos was significantly increased by treatment with lpa3 agonist but was inhibited by lpa2 agonist [75]. Accordingly, pharmacological activation of these LPA receptor subtypes may be a new strategy to enhance or inhibit erythropoiesis. 

Once EPOR binds EPO, it recruits JAK, a tyrosine kinase that can phosphorylate several tyrosine residues in EPOR. Zebrafish has three homologs of JAK: jak1, jak2a, and jak2b [76]. Overexpression of *jak2a* in zebrafish embryos increased the expression of *gata1* and hemoglobin, indicating a crucial role of jak2a in zebrafish erythropoiesis [77]. STAT proteins are transcription factors regulated by protein kinases and phosphatases. Protein tyrosine phosphatase ptpn9 was considered to regulate erythroid cell differentiation by disrupting the inhibitory complex of phosphorylated stat3, gata1, and zbp-89 in zebrafish [78]. Additionally, cytokine-inducible SH2 domain-containing protein (CISH) may suppress the STAT signaling induced by cytokines. Knockdown of *cish.a*, an ortholog of human *CISH*, led to enhanced erythropoiesis and *stat5.1* activation in zebrafish embryos, providing in vivo evidence to support the role of CISH in regulating STAT5 and erythropoiesis [79]. Additionally, a lipid kinase named PI4KA was shown to regulate JAK–STAT signaling and erythropoiesis [80]. Knockdown of *PI4KA* in mouse hematopoietic stem progenitor cells (HSPCs) impaired erythropoiesis in vitro. Loss of *pi4kaa*, the zebrafish homolog of *PI4KA***,** led to impaired erythroid differentiation [80].

Apart from the EPO–JAK–STAT pathway, regulation of the mTORC1, Rho, BMP, and TGF-β signaling pathways is also essential for zebrafish erythropoiesis. Analysis of the amino acid transporter *LAT3*, a gene upregulated during erythroblast maturation, showed that L-leucine availability controls erythropoiesis via regulating the mTORC1/4E-BP pathway [81]. Inadequate NEAA uptake induced by *lat3* deficiency and pharmacologic inhibitors of mTORC1/4E-BP pathway both led to reduced hemoglobin production in zebrafish [81]. A genome-wide association study combined with functional analyses in zebrafish revealed the critical role of the ARHGEF12–RhoA–p38 signaling pathway in erythroid regeneration [82]. During stress erythropoiesis, GATA1 upregulates the expression of ARHGEF12, which is a RhoA guanine nucleotide exchange factor that converts RhoA-GDP to RhoA-GTP [82]. The active RhoA promotes erythropoiesis through the p38–MAPK pathway [82]. Both knockout and knockdown of *arhgef12* in the zebrafish impair the erythroid differentiation, and the anemic phenotype can be rescued by treatment with p38 activator [82]. A recent study identified a new gene named BMP inhibitory factor 1 (*bif1*), which inhibits BMP signaling and is required for both primitive and definitive erythropoiesis in zebrafish [83]. Given that the BMP is known to regulate mesodermal lineage determination [84,85], it is likely that inhibition of the BMP–SMAD pathway by *Bif1* promotes the expansion of hematopoietic progenitors. Research in zebrafish also uncovered a role of splicing factor 3B subunit 1 (sf3b1) in regulating TGF-β signaling during erythropoiesis [84]. SF3B1 is a core component of the spliceosome, and its mutations can cause human erythropoietic diseases due to aberrant splicing of pre-mRNAs [86,87,88,89]. Deficiency of *sf3b1* in zebrafish embryos caused abnormal activation of the TGF-β signaling as well as cell cycle arrest and macrocytic anemia [90]. The phenotype of increased G0/G1-stage erythroid progenitors could be rescued by the inhibition of TGF-β signaling in the morphant zebrafish embryos [90].

### 3.3. MicroRNAs

A number of miRNAs, such as miR-126, miR-144, and miR-451, have been demonstrated to regulate gene expression during erythropoiesis. The miR-144/451 genomic region was first identified as a GATA-1-regulated locus essential for erythropoiesis in humans [50,91]. Zebrafish miR-451 is also clustered with miR-144, and they are in the same primary transcript, but miR-451 is about 7.5-fold more abundant than miR-144 in zebrafish erythroblasts [92]. This paradox is due to a negative-feedback loop in which miR-144 represses Dicer, which is an RNase required for the production of many miRNAs, during erythropoiesis in zebrafish [92]. Interestingly, miR-451 is refractory to the loss of Dicer because it is processed in an Ago2-dependent manner [92]. Thus, miR-451 can by-pass the global miRNA turnover during erythropoiesis to become the most abundant miRNA in erythroblasts [92]. miR-451 represses the expression of *gata2* by binding to its 3′UTR in developing erythroblasts [93]. Diminished expression of miR-451, as observed in the *meunier* mutant, impairs erythrocyte maturation in zebrafish [93]. Several other miRNAs also play a regulatory role in erythroblasts. For example, miR-26a was found to regulate erythroid proliferation and differentiation via targeting the 3’UTR of the Nemo-like kinase (NLK) in human and zebrafish models of DBA [45]. miR-200a may inhibit erythropoiesis via targeting the 3’UTR of programmed cell death 4 (PDCD4) and thyroid hormone receptor beta (THRB) [46]. miR-23a is critical for maintaining the morphology of erythrocytes, likely by targeting the protein tyrosine phosphatase SHP2 [49].

### 3.4. E3 Ubiquitin Ligases

The ubiquitin–proteasome system was first discovered in the circulating reticulocytes [94,95,96]. However, the function of ubiquitinating factors in erythropoiesis remains largely unexplored. In 2017, the E2 ubiquitin-conjugating enzyme Ube2o was reported to promote ubiquitination and degradation of ribosomal proteins in developing erythroblasts [97]. Recent studies in zebrafish led to the identification of several E3 ubiquitin ligases with critical roles in erythropoiesis. Cullin–RING E3 ligase complexes are well-known ubiquitin ligases with important roles in many biological processes [98]. Loss of *cul4a* in zebrafish embryos resulted in severely reduced erythrocyte production due to decreased expression of transcription factors *gata1*, *scl*, and *lmo2* [99]. The level of H3K4me3, a histone marker of transcription activation, in the promoter region of *gata1* was decreased after *cul4a* depletion, indicating that *cul4a* can activate the transcription of *gata1* by promoting H3K4 trimethylation [99]. 

Fish erythrocytes have nuclei, however, their nuclei are much smaller than that of the erythroid progenitors [100]. A study in zebrafish and mammal hematopoietic cell models demonstrated that Wdr26 functions as a core subunit of an E3 ubiquitin ligase complex to promote the ubiquitination and degradation of nuclear proteins during erythropoiesis [101]. Loss of *wdr26* resulted in severe anemia and enlarged nuclei in mature erythrocytes in zebrafish [101]. In developing erythroblasts, Wdr26 regulates the ubiquitination of nuclear proteins including lamins and histones [101]. Degradation of lamin B further promotes the formation of large nuclear openings, which are transiently formed on the nuclear envelope to expedite the export and degradation of nuclear proteins during erythroblast differentiation [101,102].

During the recent studies of zebrafish erythropoiesis, a number of knockout mutants have been generated (Table 1). These mutants serve as valuable genetic models for further exploring the mechanisms of erythropoiesis as well the related blood disorders.

## 4. Using Zebrafish to Study Iron and Heme Homeostasis during Erythropoiesis

Terminal erythropoiesis involves several characteristic processes including synthesis of hemoglobin, condensation and extrusion of nuclei, clearance of organelles, and remodeling of the membrane and proteome. Hemoglobin, the most abundant protein in erythrocytes, is responsible for binding and transporting oxygen in vertebrate circulation. Each hemoglobin molecule is composed of four globin chains and four heme moieties, which are iron-containing porphyrins. Accordingly, developing erythroid cells absorb massive amounts iron for the synthesis of heme and hemoglobin. A number of regulatory mechanisms for erythroid iron uptake and heme synthesis were made by studies in zebrafish, and therefore, we focus on iron and heme metabolism in this section. 

### 4.1. Iron and Heme Metabolism during Erythropoiesis

**Figure 2:**
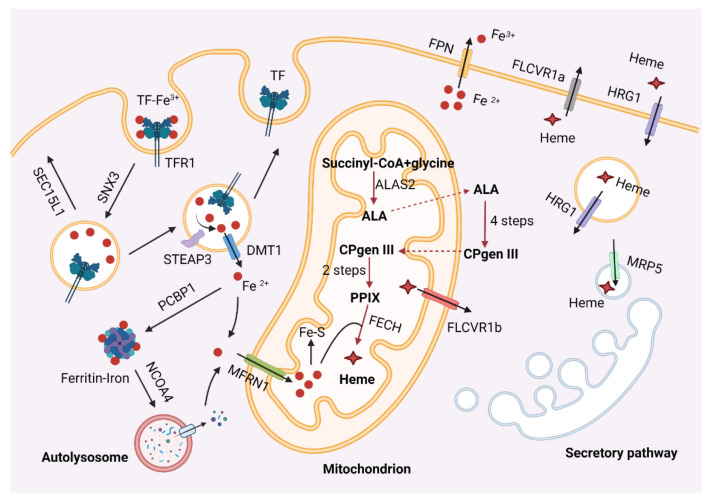
Iron and heme metabolism during erythropoiesis. Iron metabolism is shown on the left. Heme biosynthesis is shown in the middle. Heme trafficking is shown on the right. TF, transferrin; MFRN1, mitoferrin 1; DMT1, divalent metal transporter 1; PCBP1, poly r(C)-binding protein; NCOA4, nuclear receptor coactivator 4; FPN, ferroportin; FLVCR1, feline leukemia virus subgroup C receptor-related protein 1; HRG1, heme responsive gene 1; MRP5, multidrug resistance protein 5; FECH, ferrochelatase; ALA, δ-aminolevulinic acid; CPgen III, coproporphyrinogen III; PPIX, protoporphyrin IX.

In vertebrates, developing erythroblasts assimilate iron primarily through the transferrin receptor (Tfr1), which binds diferric transferrin (Tf). The Tf–Tfr1 complex is internalized into endosomes through clathrin-mediated endocytosis [106,107] (Figure 2). The Fe^3+^ in maturing endosomes is reduced to Fe^2+^ by STEAP3 [108] and exported out of endosomes by divalent metal transporter 1 (DMT1) [109]. Once inside the cytosol, iron either enters mitochondria via mitoferrin 1 (MFRN1) [110,111] for the biosynthesis of heme and iron–sulfur clusters (Fe–S) or is sequestered in the ferritin complex [112]. The rest of the Tf–Tfr1 complex undergoes sorting process in endosomes and returns to the cell membrane for the next iron uptake cycle. Given that the Tf cycle involves vesicular transport, trafficking proteins play crucial roles in this process. For example, the retromer protein SNX3 is essential for sorting the Tf–Tfr1 complex into recycling endosomes, while the exocyst component, SEC15L1, regulates the trafficking of this protein complex from recycling endosomes to the cell surface [113,114].

In addition to importing iron, animals may mobilize and utilize iron stored in ferritin for heme synthesis. Poly r(C)-binding protein (PCBP1) is a cytosolic iron chaperone responsible for transporting iron to ferritin [115]. The ferritin complex may undergo autophagic degradation, a process called ferritinophagy, to release the stored iron [116,117]. Ferritinophagy is specifically controlled by nuclear receptor coactivator 4 (NCOA4), which has been shown to be an autophagy receptor [116]. Silencing of NCOA4 led to impaired erythropoiesis in both human erythroblast cells and zebrafish embryos [118]. Ncoa4-mediated ferritin degradation is critical for macrophage iron release and erythropoiesis in mice [119]. In addition, HERC2, an E3 ubiquitin ligase, may regulate the protein level of NCOA4 in response to cellular iron levels [118].

Heme is synthesized via eight enzymatic reactions that take place in both the cytosol and mitochondria [3,120] (Figure 2). The first step, generation of δ-aminolevulinic acid (ALA) catalyzed by ALAS2, and the terminal step, formation of heme catalyzed by ferrochelatase (FECH), are two rate-limiting steps in heme synthesis. Following its synthesis, heme must be translocated from the mitochondria to the cytosol where hemoglobin resides [121,122,123] (Figure 2). In non-erythroid cells, heme also needs to be transported to other cellular compartments to be incorporated into hemoproteins [124]. Since free heme is cytotoxic, cellular heme homeostasis is strictly controlled by specific transporters and chaperones. The feline leukemia virus subgroup C receptor-related protein 1a (FLVCR1a) and its splicing variant FLVCR1b are reported to mediate the transport of heme out of the cell and the mitochondria, respectively [122,123,125]. The multidrug resistance protein 5 (MRP5) transports cytosolic heme into the secretory pathway [126], while heme responsive gene 1 (HRG1) is a heme importer localized in the plasma and endosomal membranes [127]. 

### 4.2. Recent Progress Studying Erythroid Iron and Heme Metabolism Using Zebrafish

#### 4.2.1. Iron Metabolism

Zebrafish erythroblasts rely on the Tf cycle to assimilate iron. Zebrafish has two homologs of TFR1, *tfr1a* and *tfr1b*. *tfr1a* is mainly expressed in erythroid cells, whereas *tfr1b* is ubiquitously expressed without a specific lineage preference [128]. In consistency with their expression patterns, mutations in *tfr1a* led to anemia, while morpholino knockdown of *tfr1b* did not induce defects in hemoglobin synthesis [128]. *tfr1b* may play an important role in iron uptake in neuronal systems as its silencing resulted in neurologic phenotypes [129]. In addition to Tfr1, zebrafish expresses snx3, a sorting nexin, in the erythroid tissues [113]. Knockdown of *snx3* by morpholinos induced profound iron-deficiency anemia during early development, and this defect can be complemented by supplementing non-Tf bound iron [113]. 

Work on the *frascati* (*frs*) zebrafish mutant has provided key insights into mitochondrial iron import [111]. The *frs* mutant developed hypochromic anemia, which was found to be caused by a missense mutation in a gene that encodes a solute carrier family protein (SLC25A37) [111]. This gene was shown to be a mitochondrial iron importer and named *mitoferrin* (*mfrn1*) [111]. Another mitochondrial inner-membrane protein, FAM210B, may also regulate mitochondrial iron homeostasis and heme biosynthesis in developing erythroblasts [130]. *Fam210b* is highly expressed in vertebrate erythroid tissues, and its silencing induced profound defects in heme production in both erythroblast cell models and zebrafish embryos [130]. Currently, the mechanistic role of Fam210b still remains elusive. Interestingly, the model organism *C. elegans* has a Fam210 homolog, but it does not synthesize heme, excluding the direct role of Fam210 family proteins in regulating heme synthesis [131]. 

#### 4.2.2. Heme Synthesis and Transport

Recent work in zebrafish has uncovered several new mechanisms regulating the heme synthesis genes *alas2* and *fech*. The expression of *alas2* was shown to be controlled by the hif1α/vgll4/irf2bp2 oxygen sensing pathway [103]. Under hypoxic conditions, hif1α upregulates *vgll4* expression through notch1 [103]. Vgll4 sequesters irf2bp2, a transcriptional repressor of *alas2*, and induces *alas2* expression and heme synthesis in erythroid tissues [103]. The *vgll4b* knockout fish displayed reduced erythroid heme production, leading to mitochondriopathy, increased immature erythrocytes, and reduced animal survival rate [103]. Additionally, the mitochondrial matrix peptidase, CLPX, plays dual roles in controlling the activity of ALAS. It catalyzes the incorporation of the cofactor pyridoxal phosphate into ALAS, and low-activity CLPX increases the stability of ALAS protein [132,133]. High activity of CLPX may accelerate the cofactor incorporation and ALAS degradation [133]. Morpholino knockdown of zebrafish Clpx homologs, *clpxa* and *clpxb*, results in reduced hemoglobin production and impaired erythropoiesis [132]. In addition, the activity of FECH, the final heme synthesis enzyme, may be controlled at the post-translational level. For instance, phosphorylation of FECH by the mitochondrial outer-membrane PKA is critical for FECH activity [134]. Analysis of *pinotage*, an anemic zebrafish mutant identified through a mutagenesis screen, uncovered a critical role of mitochondrial pH in controlling the activity of FECH [135]. Loss of *atpif1*, the gene responsible for the phenotype in *pinotage*, results in increased mitochondrial pH, which inhibits FECH activity [135].

The physiological roles of major heme transporters, including HRG1, MRP5, and FLVCR, have been examined in zebrafish. Transient knockdown of zebrafish *hrg1* and *mrp5* both induced defective hemoglobinization [126,127]. Hrg1 plays an essential role in recycling heme released by damaged or senescent RBCs in kidney macrophages in zebrafish [136]. Double knockout of zebrafish *hrg1a* and *hrg1b* caused heme accumulation in kidney macrophages [136]. Zebrafish has two isoforms of *flvcr1*, i.e., *flvcr1a* and *flvcr1b*. *flvcr1a* is required for the expansion of committed erythroid progenitors but cannot drive their terminal differentiation [121]. *flvcr1b* contributes to the expansion phase and is required for erythroblast differentiation [121]. The coordinated expression of *flvcr1a* and *flvcr1b* controls the cytosolic heme pool, which is critical for the regulation of erythroid progenitors and hemoglobin synthesis during terminal erythropoiesis [121].

## 5. Zebrafish Models of Erythropoietic Disorders

In humans, defects in erythropoiesis can cause various types of blood disorders such as β-thalassemia, sickle cell anemia, DBA, CDA, congenital sideroblastic anemia, microcytic hypochromic anemia, MDS, and erythropoietic protoporphyria [137,138,139]. For example, mutations in human heme synthesis genes *ALAS2* and *FECH* lead to congenital sideroblastic anemia and erythropoietic protoporphyria, respectively. Reduced globin expression and iron deficiency result in smaller erythrocytes, defined as hypochromic anemia, while iron overload can induce hemochromatosis. Additionally, abnormal structures of spectrin proteins and the erythrocyte membrane are common causes of hemolytic anemia and hereditary spherocytosis in humans. Many of these blood disorders have been characterized in zebrafish. For instance, the *sauternes*, *quem*, and *dracula* zebrafish mutants carry mutations in different heme synthesis genes, and they have been used as genetic models to study microcytic hypochromic anemia, hepatoerythropoietic porphyria, and erythropoietic protoporphyria, respectively [140,141,142]. Here, we focus on recently generated zebrafish models for CDAs, DBAs, and MDS, and these models may serve as valuable tools to develop new therapeutics for these diseases. 

### 5.1. CDAs

CDAs are characterized by reduced erythropoiesis efficiency and can be divided into several groups including CDA type I, II, III, CDA variants, and transcription-factor-related CDAs. Clinically, CDAI presents with moderate to severe giant cell anemia and abnormal erythrocyte precursors, as well as a spongy heterochromatin phenotype. This disease is caused by mutations in *CDAN1* gene. In zebrafish embryos, knockdown of *cdan1* resulted in reduced *gata1a* expression, increased *gata2a* expression, and impaired primitive erythropoiesis [143]. These results complement the data obtained from mice and suggest that *cdan1* deficiency may cause CDAI via misregulation of the erythroid transcription factors *gata1* and *gata2* [143]. 

Human CDAII is an erythroid-related disease caused by abnormal cell division, which leads to cell apoptosis, multinuclear erythroblasts, and anemias. Mutations in the anion exchanger protein *BAND 3* can cause CDAII-related dyserythropoiesis [144]. The zebrafish *retsina* mutant, which harbors a mutation in *band 3*, exhibited erythroid-specific defects in cell division and dyserythropoiesis, which resemble the symptoms of human CDAII diseases [145]. The CDAII can also be caused by bi-allelic mutations in the *SEC23B* gene that encodes a component of the COPII coatomer. Despite the fact that *SEC23B* is ubiquitous expressed in many tissues, mutations of this gene cause an erythroid-lineage-specific phenotype [146,147]. Knockdown of *sec23b* in zebrafish embryos impaired erythroid development, while knockout of *sec23b* induced more a severe phenotype and was lethal to fish within 3 weeks [148]. Expression of *sec23a* could fully rescue the phenotypes in the sec23b mutant fish [148], providing a potential way of gene therapy for treating CDAII. In addition, studies in zebrafish have provided important insights into the physiological function of heat shock cognate B (HSCB) in erythropoiesis and CDAs [149].

### 5.2. DBAs

DBAs are congenital bone marrow failure syndromes characterized by the defective development of erythroblasts. This disease has been associated with mutations and large deletions in ribosomal protein (RP) genes including *RPS7*, *RPS10*, *RPS17*, *RPS19*, *RPS24*, *RPS26*, *RPS29*, *RPL5*, *RPL11*, *RPL26*, and *RPL35A* as well as *GATA1* [138,150]. Elevated activity of the tumor suppressor p53 was also observed in DBA patients [151]. Zebrafish has been used for pathogenesis studies and drug screens related to DBAs because it has advantages in high-throughput genetic screens and chemical biological studies. Chemical screens in zebrafish have identified a number of compounds such as L-leucine, sotatercept, and trifluoperazine as potentially effective drugs for DBAs [152].

In recent years, the physiological roles of many ribosomal genes in zebrafish erythropoiesis have been characterized. Transient knockdown of these genes with morpholinos often leads to defective erythropoiesis in zebrafish. For instance, *rpl5* is required for both primitive and definitive erythropoiesis processes in zebrafish embryos [153]. *rpl18*-deficient zebrafish embryos developed anemia with reduced *gata1* and globin expression [154]. In addition, silencing of *rpl27* and *rps27* both resulted in reduced hemoglobin expression and impaired erythrocyte production [155].

Increased activity of the tumor suppressor p53 is observed in DBA patients [151]. In zebrafish, loss of *rps9* and *rpl10a* both led to impairment of erythroblast maturation and anemia in a p53-dependent manner [156,157]. Similarly, *rpl18* deficiency increased the activities of both p53 and jak2–stat3 pathways, which could be rescued by inhibitors of jak2 or stat3 phosphorylation [154]. *rps29* mutant zebrafish embryos, another model of DBAs, showed a p53-dependent anemia, and this phenotype could be rescued by calmodulin inhibitors [14]. In addition, ribosomal dysfunction in DBA patients can activate Nemo-like kinase (NLK), which is hyperactivated in committed erythroid progenitors. Knockdown of *rps19* in zebrafish embryos induced anemia, which could be rescued by metformin injection [45]. Further studies demonstrated that metformin induced the upregulation of miR-26a, which targets NLK for degradation, to improve erythropoiesis in human cells [45].

### 5.3. MDSs

MDSs are a group of malignant bone marrow disorders, which are associated with ineffective erythropoiesis. The current standard of the classification of MDS is based on patients of refractory anemia (RA) with or without ringed sideroblasts. Recent studies in zebrafish mainly focus on two subtypes of MDSs, including refractory anemia with ring sideroblasts (RARS) and MDS associated with isolated del(5q). Splicing factor 3B subunit 1 (SF3B1) is one of the most prevalently mutated factors in RARS subtype of MDS [90]. Loss-of-function mutations in zebrafish *sf3b1* induce macrocytic anemias with enhanced TGF-β signaling, and inhibition of TGF-β signaling pathway can release the G0/G1 block of erythroid progenitors in the mutants [90]. Thus, combined medication with TGFβ superfamily inhibitors and known SF3B1-modulating drugs may be a more effective method to treat MDS patients [90]. The other major subtype of MDS, del(5q) MDS, includes deletion of the *RPS14* gene, which results in macrocytic anemia [158]. The *rps14*-knockout zebrafish has been used to screen for new drugs for the treatment of del(5q) MDS [159]. This screen identified inhibitors of matrix metalloproteinase 9 (MMP9) that significantly rescued the erythroid defects in rps14-deficient zebrafish [159]. Further study demonstrated that mmp9 was upregulated in rps14-deficient cells to inhibit erythropoiesis via enhanced TGF-β signaling [159]. This work suggests that MMP9 inhibitors may serve as therapeutic agents for patients with del(5q) MDS. It was reported that mutations in DEAD-box helicase 41 (DDX41) was also associated with blood disorders including MDS [160]. Functional analysis of *ddx41* in zebrafish embryos revealed that it was required for the expansion and differentiation of erythroid progenitors [161], and these results promoted the understanding of hematologic malignancies induced by mutations in DDX41. 

## 6. Conclusions

The zebrafish is an excellent vertebrate model to study erythropoiesis, because it has many advantages for genetic studies and the processes of erythropoiesis are highly conserved between fish and mammals. This model animal permits forward genetic screens, reverse genetic studies, large-scale chemical screens, and targeted gene function studies. Studies on zebrafish have provided important insights into the fundamental understanding of erythropoiesis, especially in iron and heme metabolism and regulation of erythropoiesis. The establishment of zebrafish models of blood-related diseases provides valuable resources for developing new therapeutic treatments of these diseases.

## Figures and Tables

**Table 1:** Recently generated zebrafish mutants with defective erythropoiesis.

Mammalian Genes	Zebrafish Mutants	Phenotypes in Erythropoiesis	References
*VGLL4B*	*vgll4b−/−*	Abnormal erythrocytes	[103]
*WDR26B*	*wdr26b−/−*	Anemia, impaired nuclear condensation, susceptible to hypoxia	[101]
*CUL4A*	*cul4a−/−*	Impaired erythroid differentiation, reduced *gata1* level	[99]
*P2Y12*	*p2y12−/−*	Excessive erythropoiesis, increased globin expression	[62]
*HMGCS1*	*hmgcs1−/−*	Decreased number of mature RBCs, reduced *gata1* expression	[104]
*MIR-144*	*miR-144−/−*	Enlarged nuclei, impaired chromatin condensation, impaired erythrocyte maturation	[92,105]

## Data Availability

Not applicable.
